# Diet Quality, Metabolic Syndrome, and Nativity Status: Elucidating Metabolic Advantage and Disadvantage Among Non-US-Native and US-Native Populations Using NHANES Data (2013–2018)

**DOI:** 10.3390/nu17020215

**Published:** 2025-01-08

**Authors:** Danyel I. Smith, Eren Sakarcan, Lucile Adams-Campbell, Chiranjeev Dash

**Affiliations:** 1 Office of Minority Health and Health Disparities Research, Georgetown Lombardi Comprehensive Cancer Center, Georgetown University, 1010 New Jersey Ave. SE, Washington, DC 20003, USA; lla9@georgetown.edu (L.A.-C.); cd422@georgetown.edu (C.D.); 2 School of Medicine, University of South Carolina, 6311 Garners Ferry Rd., Columbia, SC 29209, USA; eren.sakarcan@uscmed.sc.edu

**Keywords:** metabolic syndrome, nativity status, diet quality, health disparities

## Abstract

Background/Objectives: Nutrient-poor diet quality is a major driver of the global burden of metabolic syndrome (MetS). The US ranks among the lowest in diet quality and has the highest rate of immigration, which may present unique challenges for non-US-native populations who experience changes in access to health-promoting resources. This study examined associations among MetS, nativity status, diet quality, and interaction effects of race–ethnicity among Hispanic, Asian, Black, and White US-native and non-US-native adults. Methods: We examined data from 5482 adult participants (≥20 years of age) in the National Health and Nutrition Examination Survey (2013–2018). MetS (per the ATP III panel guidelines) was assessed continuously (MetS z-score) and dichotomously. Dietary recalls were used to compute HEI-2015 scores. Nativity status and sociodemographic variables were assessed. Age-adjusted and multivariate-adjusted logistic regressions were conducted to examine the associations between nativity status and MetS and interaction effects by race–ethnicity. Results: Non-US-native participants displayed more guideline-adherent diet quality (55.23% vs. 49.38%, *p* < 0.001) compared to their US-native counterparts—even when stratified by racial–ethnic groups. US-native participants had larger waist circumferences and elevated triglyceride levels. Non-US-native Black Americans had a 60% lower risk of having MetS even after adjusting for diet quality (OR: 0.39, 95% CI: 0.17, 0.88) compared to their US-native counterparts. For MetS components, non-US-native Asian participants reported a lower risk for dyslipidemia, while non-US-native multiracial participants had higher triglycerides. Conclusions: Non-US-native groups display better diet quality compared to their US-native counterparts. However, the findings suggest that diet quality alone does not account for nativity-related cardiometabolic disparities, particularly in US-native Black Americans, thus necessitating interventions targeting the social determinants of health.

## 1. Introduction

Nutrient-poor diet quality and physical inactivity are major drivers of the global burden of metabolic syndrome (MetS), which impacts 12.5–31.4% of people worldwide [1]. The components of MetS (i.e., central adiposity, hypertension, dyslipidemia, low HDL cholesterol, and glucose intolerance) singularly impact the global burden of disease, with nearly 4 in 10 adults affected by hypertension and 3 out of 10 adults impacted by central obesity [1]. Across various dietary patterns—including the Mediterranean diet, Dietary Approaches to Stop Hypertension (DASH) diet, and plant-based and high-protein diets—diets rich in fruits and vegetables, with limited intake of animal products and alcohol, and low consumption of processed foods can improve metabolic outcomes [2], including a reduction in cardiovascular disease incidence [3], decreased blood pressure, reduced mortality, regulated lipid levels, and reduced diabetes incidence. Contrarily, low diet quality is associated with an increased risk for adverse levels of metabolic factors, including overweight/obesity and elevated glucose levels [4]. Metrics of dietary quality include the healthy eating index (HEI) score, which indicates adherence to the Dietary Guidelines for Americans.

Dietary quality is intrinsically linked with dietary patterns, which are cultural byproducts that are constrained by environmental affordances (including physical and sociocultural factors). A recent report of data from the Global Dietary Database indicated that higher-income countries report guideline-adherent (per the Alternate Healthy Eating Index (AHEI)) consumption of healthy dietary components (e.g., fruits) but suboptimal consumption of less healthy components (e.g., red/processed meats) compared to lower-income countries [5]. Notably, the US was among the countries with the lowest dietary quality in the world [5]. In addition to environmental constraints, dietary quality is shaped by several social determinants of health (e.g., housing and transportation), which differentially influence access to health-promoting resources (e.g., grocery stores). Immigration status is a social determinant of health [6], which may influence diet quality due to shifts in environmental (e.g., housing and food access) and social affordances (e.g., immigration policies and healthcare access).

A record-reaching 44.8 million immigrants comprised the US population in 2018—accounting for 13.7% of the US population, according to the Pew Research Center [7]. This exponential growth has substantial implications for population health as immigrants interface with new physical (e.g., neighborhood access), sociocultural (e.g., racialized culture), and environmental affordances (e.g., social determinants of health), which may increase vulnerability to negative health outcomes [6]. Metabolic risk factors (e.g., diet quality) are sensitive to changes in environmental affordances, such as food quality and availability [8].

Among immigrant populations in the US, MetS and metabolic risk factors are highly prevalent [9,10]. For example, hypertension rates are higher among immigrants from Southeast Asia, Africa, Mexico, Central America, and the Caribbean compared to immigrants from Europe [9]. Additionally, immigrants from Mexico/Central America and the Caribbean, as well as the Indian subcontinent, displayed the highest prevalence of overweight/obesity, per 2010–2016 National Health Interview Survey data [11]. Taken together, these data suggest that country of origin may influence exposure to metabolic risk factors and MetS-related health care (e.g., prescriptions) [9]. However, when comparing the health outcomes of migrant populations to their US-native counterparts, the results indicate a health advantage for immigrant populations, frequently cited as the “immigrant health paradox” in which non-US-native individuals experience better health outcomes [12]. For example, data from the RAND American Panel of Life found evidence to support this paradox for certain disease outcomes. Specifically, non-US-native respondents displayed lower rates of depression, nerve pain, and obesity compared to their US-native counterparts [13]. Additionally, non-US-native Hispanic immigrants displayed a “metabolic advantage” over their US-native counterparts. Carabello and Wolfson [14] examined metabolic health indicators among US-native Mexican Americans, non-US-native Mexican Immigrants, and US-native White Americans using NHANES 2013–2018 data. Their results showed that non-US-native Mexican immigrants had a lower unadjusted prevalence for MetS compared to their US-native counterparts [14]. Similarly, US-native Hispanic and Black Americans demonstrated higher levels of metabolic dysregulation compared to non-US-native Hispanic immigrants, whose metabolic risks were comparable to White Americans [15].

Other studies contest the generalizability of the immigrant health paradox due to its omission of the intersectionality of race and socioeconomic status in the immigrant experience [12]. For example, national data suggest that US- and non-US-native Hispanic individuals have similar overall health profiles (including physical functioning, depression, and metabolic dysregulation), irrespective of nativity status [15]. Interestingly, non-US-native Hispanic immigrants display similar levels of systemic inflammation (i.e., C-reactive protein) to US-native Black Americans, suggesting similarities in biophysiological responses to metabolic risk factors (e.g., stress) [15]. Social contextual factors implicated by race and socioeconomic status (e.g., unemployment) may increase food insecurity, thereby influencing the diet quality of immigrant populations [11]. In sum, immigrant metabolic health outcomes are complex and may differ based on new affordances to health-promoting resources.

Although nutrient-poor diet quality is commonplace due to heightened globalization [1], countries with higher rates of MetS (e.g., the US) may differentially afford resources that increase the risk for MetS for native and non-native populations—yielding nativity-related disparities in cardiometabolic health. While the relationship between diet quality and MetS is well documented [16], only one study, to our knowledge, has examined the relationship between diet quality and cardiometabolic health and the role of nativity status [11]. Moreover, prior studies have focused on singular ethnic group comparisons (e.g., US-native Mexican immigrants vs. US-native Mexican Americans [13,14]) and have not included a diverse group of immigrants to assess heterogeneity in immigrant health outcomes. There is a need for disaggregated, granular analyses by nativity status and country of origin to guide resource allocation and optimize intervention strategies for specific subpopulations [11]. In recognizing the interplay of nativity status and race/ethnicity, policymakers may be better equipped to design culturally sensitive public health initiatives that address the unique social determinants of health faced by immigrant communities, such as language barriers or food accessibility. This approach not only improves the precision of public health data but also fosters equity by elucidating and addressing the specific needs of underrepresented immigrant groups. The goal of this study was to examine associations between MetS and nativity status, as well as interactions among MetS, nativity status, and race/ethnicity. Based on the prior literature, we expected that non-US-native individuals would display lower rates of MetS and better diet quality than their US-native counterparts across racial–ethnic groups. Moreover, we expected diet quality to account for any differences in MetS prevalence observed between US-native and non-US-native individuals.

## 2. Materials and Methods

**Figure 1:**
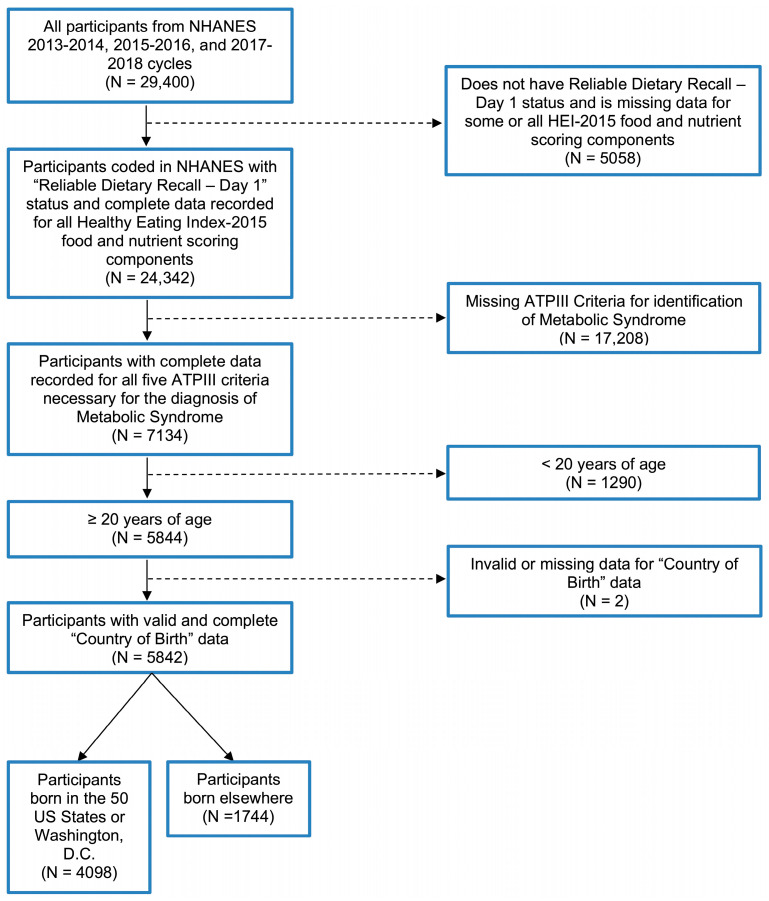
Study population exclusion criteria flowchart.

We examined data from the National Health and Nutrition Examination Survey (NHANES) 2013–2018 cycles based on the following eligibility criteria: (a) presence of complete dietary recall, (b) complete data for ATP III criteria for MetS, (c) ≥20 years of age, and (d) complete data for “country” of birth. This yielded a final sample size of 5482 participants. See Figure 1 for exclusion criteria.

### 2.1. Metabolic Syndrome

MetS was defined as three of the following clinical criteria per US National Cholesterol Education Program Adult Treatment Panel III guidelines: waist circumference (>40 inches, male; >35 inches, female), serum triglycerides (≥150 mg/dL), HDL cholesterol (<40 mg/dL, male; <50 mg/dL, female), systolic blood pressure (>130 mmHg) or diastolic blood pressure (>85 mmHg), and fasting plasma glucose (≥100 mg/dL). MetS was analyzed using a dichotomous (i.e., 0, does not meet clinical cutoff; 1, does meet clinical cutoff) and continuous score (i.e., MetS z-score).

### 2.2. Dietary Quality

NHANES utilizes the USDA’s computer-assisted, 24 h dietary recall to assess dietary quality. Using a multiple-pass method, two consecutive 24 h recalls were captured. Healthy Eating Index (HEI) 2015 scores were calculated from recall data to assess dietary quality in accordance with the 2015 Dietary Guidelines for Americans (DGA). Weighted means for HEI-2015 scores were computed using the population ratio method [17] and assessed dietary quality for the total sample, whereas the simple scoring method [17] was used to assess dietary quality at the individual level. HEI-2015 scores ranged from 0 to 100, with higher scores equating to better diet quality and greater adherence to the 2015–2020 DGA guidelines. Quintiles were also computed for logistic regression analyses.

### 2.3. Nativity Status

The demographic section of NHANES assessed country of birth. Participants self-reported country of birth. Participants who indicated the US as their country of birth were coded as US-native individuals, and those who were born elsewhere were coded as non-US-native individuals.

### 2.4. Sociodemographics

Data on age, sex, race/ethnicity (Mexican American, Other Hispanic, non-Hispanic White, non-Hispanic Black, non-Hispanic Asian, and not listed [including multi-racial]), nativity (US native, non-US native), years of US residency, education level, marital status, and annual household income were captured in NHANES demographic section. Only age, sex, race/ethnicity, nativity, and education level were entered into analyses as covariates.

### 2.5. Statistical Analysis

Data from the six-year sampling interview, physical exam, and dietary recall day 1 were used for combined dataset analyses. All analyses were conducted using SAS Studio User Software *v.3.8), and weighted chi-square tests were conducted to assess significant differences in proportions for categorical variables. One-way ANOVA models were used to assess mean differences in continuous variables. A series of logistic regressions were conducted to assess the association between MetS and nativity status, including crude, age-adjusted, and multivariate-adjusted models. Two multivariate-adjusted logistic regressions were conducted, accounting for (1) age, race/ethnicity, sex, and education level and (2) age, race/ethnicity, sex, education, and diet quality (i.e., HEI-2015 total score quintiles). Interaction effects of race/ethnicity, sex, and education on the relationship between nativity status and MetS were assessed. Further, age- and multivariate-adjusted models were stratified by race/ethnicity. Significant interactions were further probed by calculating odds ratios by slicing the least square means analyses of the interaction terms by the variables involved in the interactions.

## 3. Results

Non-US-native participants comprised 17% of the sample (*n* = 1744), with most residing in the US between 20 and 30 years. The participant demographics regarding nativity status can be found in Table 1. Regarding race–ethnicity, US-native participants were more likely to identify as Black or White Americans compared to other racial–ethnic groups. US-native participants displayed a higher prevalence of obesity (per BMI status) compared to non-US-native participants (*p* < 0.0001). US-native participants were more likely to have at least some college education (*p* < 0.0001) and earn USD 45,000–USD 74,999 in yearly household income (*p* < 0.0001).

Regarding components of MetS, non-US-native participants had more elevated triglycerides (*p* = 0.003) and lower HDL cholesterol (*p* = 0.04) compared to their US-native counterparts. US-native participants displayed larger waist circumferences (*p* < 0.0001) compared to non-US-native participants. There was no statistically significant difference in MetS prevalence between groups (*p* = 0.33), as US-native and non-US-native participants had similar rates of MetS at 37.9% and 36.3%, respectively. See Table 2.

**Figure 2:**
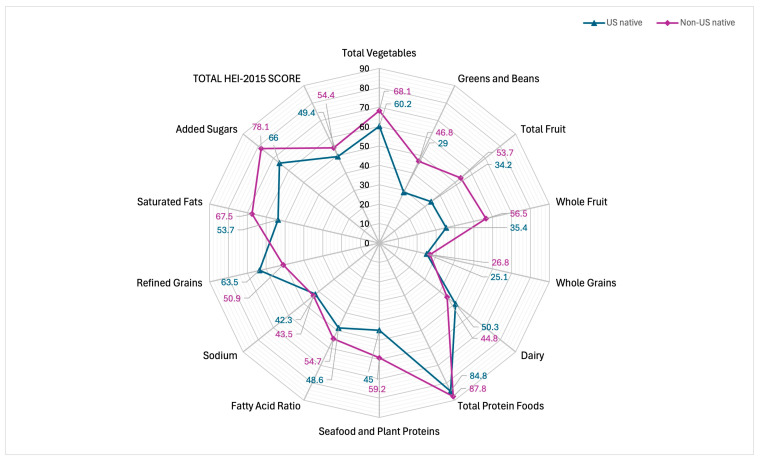
Comparing individual-level Healthy Eating Index 2015 scores of US-native and non-US-native participants. A radar plot of HEI-2015 scores for US-native (49.4%) and non-US-native (54.4%) participants.

Regarding diet quality, non-US-native participants demonstrated more DGA guideline-adherent diets (55.23%) compared to US-native participants (49.38%; *p* < 0.001). Both US-native and non-US-native participants showed the greatest adherence to proteins (84.8% and 87.8%, respectively) and the lowest adherence among whole-grain food groups (25.1% and 26.8%, respectively). See Table 3. Figure 2 depicts a radar plot to visualize adherence to DGA guidelines for US-natives and non-US-native participants. Dietary components plotted closer to the center reflect less guideline adherence, whereas dietary components plotted closer to the perimeter reflect more guideline adherence. Similar results were found when stratifying by race/ethnicity. Non-US-native participants reported better diet quality compared to their US-native counterparts for Mexican American, (*p* < 0.0001), Other Hispanic (*p* = 0.007), White (*p* < 0.0001), Black (*p* < 0.001), Asian, (*p* = 0.003), and Other/Multiracial (*p* = 0.02) groups. See Table 4.

### Interactions Among Diet Quality, MetS, and Nativity Status

**Figure 3:**
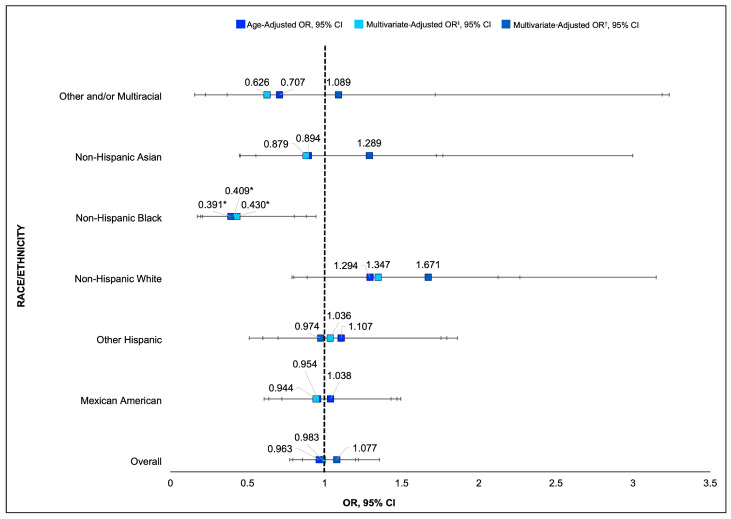
Combined forest plot showing age-adjusted and multivariate-adjusted odds ratios and 95% CIs for MetS and nativity by race/ethnicity. ‡ Multivariate-adjusted OR—controlling for age, race/ethnicity, sex, and education. † Multivariate-adjusted OR—controlling for age, race/ethnicity, sex, education, and HEI-2015 score. * *p* < 0.05 indicates a statistically significant difference in odds of developing MetS compared to US-native participants of the same race/ethnicity.

When examining interactions by MetS and race/ethnicity, age-adjusted associations revealed that non-US-native, non-Hispanic Black individuals were 59% less likely to have MetS compared to their US-native counterparts (OR: 0.41, 95% CI: 0.21–0.80). This effect remained significant after adjusting for diet quality (OR: 0.39, 95% CI: 0.17–0.88), suggesting that diet does not fully account for differences in MetS prevalence among US-native and non-US-native non-Hispanic Black individuals (See Table 5 and Figure 3).

Similar results were found when examining the clinical components of MetS. Non-US-native, non-Hispanic Black individuals were 46% less likely to have a waist circumference (OR: 0.54, 95% CI: 0.33–0.94) and 48% less likely to have blood pressure (OR: 0.52, 95% CI: 0.29–0.95) meeting the MetS clinical diagnosis criteria. The interactive effect of race/ethnicity on the relationship between MetS prevalence and nativity status was not significant for any other ethnic group. However, the results indicated significant differences in the clinical components of MetS by nativity status within racial/ethnic groups. See Table 6. Non-US-native Asian participants were 69% less likely to have HDL levels meeting the MetS clinical criteria (OR: 0.31, 95% CI: 0.14–0.70) compared to their US-native counterparts. Non-US-native multiracial participants were 4.97 times as likely to have triglyceride levels exceeding the MetS clinical cutoffs compared to US-native multiracial participants (OR: 4.97, 95% CI: 1.14–21.54), suggesting increased risk for cardiovascular disease among multiracial non-US-native participants.

## 4. Discussion

MetS is a global health issue implicated in the worldwide burden of cardiovascular disease. This cross-sectional study utilized NHANES 2013–2018 data to examine the associations of MetS, nativity status, and dietary quality within a sample of 5482 participants. The results suggest greater metabolic dysfunction among US-native participants compared to non-US-native participants. These findings are consistent with evidence supporting the immigrant health paradox, such that non-US natives displayed better physical health outcomes compared to their non-US-native counterparts. In a nationwide study, US-native respondents were 41% more likely to have *any* health condition and 32% more likely to have multimorbidity compared to non-US-native respondents [13]. Moreover, being born in the US was associated with 60% greater odds of having obesity compared to respondents born outside of the US [13].

Three rationales for this paradox pervade the immigrant health literature: cultural buffering/acculturation, social/neighborhood-level factors, and methodological gaps. Firstly, acculturation-based frameworks assert that cultural value systems protect (or provide a buffer against) newly migrated populations against negative health outcomes extending from social and economic disadvantage within the new host country. This assertion relies on individual-level factors (e.g., behaviors and cultural values), which are decontextualized from social and physical environments [18]. Secondarily, recent research posits that ecological factors in the new country (e.g., neighborhood environment and networks) influence cultural norms learned from the country of origin [18], leading individuals to gradually shift behaviors over time. Lastly, the salmon bias hypothesis (i.e., less-healthy immigrants travel back to their native country to seek treatment for diseases) is frequently cited to explain methodological gaps in obtaining comprehensive data from immigrant populations. While much attention has been paid to this healthy immigrant “advantage”, the comparative disadvantage of US-native subpopulations should be further explored.

More granular analyses of interactions of MetS, nativity status, and race and ethnicity revealed a metabolic “advantage” for non-US-native participants and a corresponding “disadvantage” for US-native participants. Non-US-native Black participants had lower rates of MetS compared to US-native participants, suggesting that US-native Black Americans may interface with health determinants that increase the risk for metabolic dysfunction. While poor diet quality among US-native Black Americans (compared to their non-US-native counterparts) partially explains the metabolic *dis*advantage of US-native Black Americans [12,19], diet quality alone does not explain the cardiometabolic disparities among US-native Black Americans.

One plausible explanation for the US-native metabolic *dis*advantage among Black Americans relates to the racialized social system in the US and subsequent minority stress [20]. Given that the US social order entangles the allocation of resources with the social category of race, environmental affordances (including structures [e.g., policies]) can create a lived context that is inequitably health-compromising for Black Americans born within the country. Marginalization (e.g., discrimination) and disenfranchisement from health-promoting resources can induce psychosocial stress, a known metabolic risk factor [21]. Discrimination is a prominent, multilevel psychosocial stressor that can facilitate the development of psychologically protective but physiologically harmful mechanisms that implicate MetS diagnosis and severity [22]. Discrimination and the anticipation of a discriminatory event (i.e., vigilance) have been linked with increased waist circumference. Black women who reported high vigilance had a 3.9 cm larger waist circumference compared to women who did not report experiencing vigilance [23]. Behaviorally, lifetime discrimination is associated with higher consumption of unhealthy foods [24], which downregulates the release of cortisol in the short term [21] but can increase the risk for MetS and poor health outcomes in the long term [25]. Black Americans may also respond to systemic disinvestment and poor access to critical determinants of health (e.g., chronic poverty) with an unrelenting determination to succeed and high-effort coping (i.e., John Henryism), which has been linked with a high incidence of MetS among Black young adults [26]. Multilevel strategies to improve metabolic profiles among US Black Americans are intrinsically linked with access to welfare-promoting resources (e.g., stress-reducing activities and recreational facilities).

Among Asian Americans, a similar immigrant health advantage was observed. Non-US-native Asian Americans had a lower risk of dyslipidemia compared to their US-native counterparts. This is partially consistent with prior research that found a lower risk of prevalent type 2 diabetes and prediabetes among non-US-native Asian Americans who have lived in the US for <15 years; however, this metabolic advantage wanes with increasing years in the US (i.e., >15 years), suggesting that cumulative exposure to environmental affordances within the US increases disease risk among Asian immigrants [27]. Indeed, immigration-related stressors, such as language and ethnic discrimination, may facilitate self-regulatory coping behaviors (e.g., emotional eating) that increase MetS risk across immigrant populations, including Asian, Hispanic, and Afro-Caribbean groups [11]. Although our study findings did not provide evidence of an association between nativity status, diet quality, and MetS for Mexican and Other Hispanic participants, prior studies have found nativity-related cardiometabolic disparities amongst Hispanic populations. In particular, a systematic review of the literature on MetS among Hispanic immigrants in the US found that US-native Hispanic Americans displayed higher rates of hyperglycemia and hypertension compared to non-US-native Hispanic immigrants [28]. However, within-immigrant analyses reveal that immigrants who have resided in the US for a longer duration (>10 years) display greater metabolic dysfunction compared to those residing in the US for fewer years (<5 years) [29]. Notably, US-native Hispanic Americans tend to display similar metabolic profiles to US-native Black Americans, which can be explained by differences in socioeconomic status, education, and exposure to stress [15]. Moreover, the weakened metabolic advantage of non-US Hispanic immigrants stemming from longer durations in the US could be improved by addressing disparities in education, income, and food security [14]. In sum, the results suggest that addressing the social determinants of health (e.g., education and socioeconomic status) can improve the metabolic profiles of Asian and Hispanic groups, irrespective of nativity status.

Lastly, we found that non-US-native multiracial participants displayed a higher odds of having triglyceride levels meeting clinical cutoffs for MetS compared to US-native multiracial participants. One plausible explanation for this unexpected finding relates to the group composition. The multiracial category was an aggregate of respondents from any region not specified on the survey and any respondents from a combination of national or racial backgrounds, thereby increasing difficulty in ascertaining whether metabolic risk factors are attributable to the process of immigration and exposure within the US or biological risk factors related to geographic regions or ancestry that may predispose individuals to MetS. In 2022, 20% of immigrants to the US reported being of a racial group other than Asian, White, Black, American Indian/Alaska Native, or Native Hawaiian or Pacific Islander [30]. In the same study, another 21% reported having two or more races [30], highlighting the need for future research to disaggregate and stratify analyses by country background to ascertain factors implicating metabolic risk among multiracial, multiethnic immigrants. Taken together, these results suggest a metabolic advantage among non-US-native immigrants and a corresponding disadvantage among US-native counterparts.

One recommendation to improve the diet quality of US-native populations involves leveraging existing federal infrastructure to address food and nutrition insecurity (e.g., the Supplemental Nutrition Assistance Program) to incentivize purchasing and consuming food components that show low DGA adherence (e.g., whole grains) [31]. To ameliorate the noted metabolic disadvantage of US-native Black Americans, researchers should augment existing behavioral interventions with stress management education to aid in mitigating the prolonged release of cortisol hormones, a known metabolic risk factor [32]. Indeed, Cox et al. (2015) [33] found significant weight loss and reduced cortisol levels when pairing education of stress management techniques with a behavioral weight loss intervention (i.e., the Diabetes Prevention Program).

### Strengths and Limitations

The findings from this study should be interpreted within the context of its limitations and strengths. Firstly, neither years in the US nor age at immigration (not available in the dataset) were included in the multivariate-adjusted models, which, when included in analyses in prior studies, revealed a trend resembling a dose-dependent effect, such that physical health outcomes worsened with increasing years in the US in certain ethnic groups, including Mexican Americans and Asian Americans [27]. Additionally, analyses were independently adjusted for social identities (e.g., gender, race, income level, and education) and did not account for intersectional identities (i.e., race × gender). Future studies should examine how mutually constitutive social structures impacting sex, race, and class (e.g., sexism, racism, and classism) may synergistically produce inequity among US-native [34,35] and non-US-native [18] individuals to shape health trajectories.

Despite the noted limitations, this study has several strengths. We leveraged a large, nationally representative sample of US- and non-US-native individuals (*N* = 5842) from NHANES 2013–2018 data, yielding optimal statistical power and the generalizability of the results to US adults. By conducting granular analyses of interactions by race/ethnicity and nativity status, this study was the first to effectively pinpoint nativity-related cardiometabolic disparities of adults residing in the US. Lastly, our findings uniquely provide insight into specific dietary components (e.g., whole grain consumption) of both US-native and non-US-native respondents, allowing researchers and public health professionals to identify specific intervention targets for nutrition education and dietary change.

## 5. Conclusions

Using NHANES 2013–2018 data, we observed better diet quality and more adherence to DGA guidelines amongst non-US-native Hispanic (including Mexican and Other Hispanic groups), Black, Asian, and White groups. We also observed an overall metabolic advantage among non-US-native groups and a corresponding metabolic disadvantage among US-native groups. US-native Black Americans displayed higher rates of MetS—above and beyond diet quality—compared to their non-US-native counterparts, suggesting that US-native Black Americans are inequitably exposed to environments and resources that increase metabolic risk (e.g., psychosocial stress). The findings for differences by nativity status and MetS components were mixed, with non-US-native Asian Americans reporting a metabolic advantage (compared to their US-native counterparts) and non-US-native other multiracial Americans reporting a metabolic disadvantage (compared to their US-native counterparts). No significant results were found by nativity status for Hispanic Americans (Mexican and Other Hispanic). The findings from this study demonstrate the need for targeted interventions amongst US-native and non-US-native groups that address the social determinants of health known to impact diet quality, like education and socioeconomic status, in order to improve metabolic profiles.

## Figures and Tables

**Table 1:** Baseline population and socioeconomic characteristics of final study sample (*N* = 5842).

Variable	*N* (%)	US-Native (*n* = 4098)	Non-US-Native (*n* = 1744)	*p*
Age (Years) [*n* (%)] *				<0.0001
20–29	921 (17.9%)	730 (88.7)	191 (13.2)	
30–39	983 (17.1%)	641 (74.5)	342 (25.5)	
40–49	952 (17.3%)	608 (77.8)	344 (22.2)	
50–59	1033 (19.5%)	679 (82.4)	354 (17.6)	
60–69	1061 (16.9%)	721 (87.4)	340(12.6)	
70–79	580 (7.7%)	435 (85.8)	145 (14.2)	
≥80	312 (3.6%)	284 (93.8)	28 (6.2)	
**Sex**				0.6827
Male	2849 (49.2%)	2004 (82.3)	845 (17.7)	
Female	2993 (50.8%)	2094 (82.7)	899 (17.3)	
Race/Ethnicity				<0.0001
Mexican American	856 (8.7%)	345 (43.75)	511 (56.2)	
Other Hispanic	647 (6.0%)	225 (39.0)	422 (61.0)	
Non-Hispanic White	2270 (66.2%)	2163 (95.0)	107 (5.0)	
Non-Hispanic Black	1193 (10.2%)	1086 (90.9)	107 (9.1)	
Non-Hispanic Asian	653 (4.9)	81 (13.2)	572 (86.8)	
Other race—including multiracial	223 (3.8)	198 (87.4)	25 (12.6)	
Years of Residency in the US *				<0.0001
<1	46 (0.60)	--	46 (0.60)	
≥1, <10	297 (3.3)	--	297 (3.3)	
≥10, <20	432 (4.8)	--	432 (4.8)	
20+	902 (8.3)	--	902 (8.3)	
Refused	26 (0.25)	--	26 (0.25)	
Did not know	41 (0.55)	--	41 (0.55)	
BMI Category				<0.0001
Underweight		67 (1.6)	21 (1.1)	
Normal weight		1016 (26.3)	536 (30.4)	
Overweight		1226 (30.9)	671 (38.2)	
Obesity		1789 (41.2)	516 (30.3)	
Education Level				<0.0001
<12 years	1213 (13.4)	618 (15.1)	595 (34.1)	
High school, GED, or equivalent	1304 (23)	1013 (24.7)	291 (16.6)	
Some college	1828 (32.1)	1481 (36.1)	347 (19.9)	
College or above	1495 (31.5)	985 (24.0)	510 (29.2)	
Refused or did not know	2 (0.01)	1 (0.02)	1 (0.05)	
Household Income				<0.0001
<USD 20,000	990 (11.1)	744 (77.7)	305 (22.3)	
USD 20,000–44,999	1601 (22.8)	1125 (78.4)	478 (21.6)	
USD 45,000–74,999	1089 (20.7)	813 (86.6)	276 (13.4)	
USD 75,000–99,000	549 (11.9)	409 (87.1)	140 (12.9)	
USD 100,000+	1032 (25.4)	712 (86.5)	320 (13.5)	
Missing, refused, or did not know	329 (4.13)	179 (66.4)	150 (33.6)	

* Years of residency in the US were only captured for non-US-native respondents.

**Table 2:** Descriptive statistics for MetS and associated clinical components by nativity status.

	US-Native (*n* = 4098)	Non-US-Native (*n* = 1744)	*p* ‡
Clinical MetS Components (Mean ± SE) **			
Fasting plasma glucose (mg/dL)	107.661 ± 0.602	109.419 ± 1.022	0.159
HDL cholesterol (mg/dL)	55.063 ± 0.429	53.509 ± 0.626	0.045 *
Male	49.312 ± 0.513	47.750 ± 0.692	0.109
Female	60.613 ± 0.668	59.187 ± 0.935	0.172
Triglycerides (mg/dL)	113.143 ± 1.819	127.725 ± 4.598	0.003 *
Mean arterial pressure (mmHg)	87.417 ± 0.269	87.472 ± 0.426	0.907
Systolic blood pressure	122.245 ± 0.347	120.899 ± 0.622	0.060
Diastolic blood pressure	70.003 ± 0.338	70.758 ± 0.501	0.142
Waist circumference (cm)	101.268 ± 0.419	95.492 ± 0.561	<0.0001 *
Male	103.078 ± 0.513	97.709 ± 0.754	<0.0001 *
Female	99.521 ± 0.546	93.306 ± 0.746	<0.0001 *
MetS z-score	−0.608 ± 0.064	−0.684 ± 0.100	0.473
Male	−0.712 ± 0.087	−0.716 ± 0.124	0.977
Female	−0.509 ± 0.085	−0.653 ± 0.144	0.347
Categorical MetS designation [*n* (%)] †			0.336
Does not have MetS	2545 (62.1)	1111 (63.7)	
Has MetS	1553 (37.9)	633 (36.3)	
Categorical MetS score [*n* (%)] †			0.324
0	200 (79.9)	90 (20.1)	
1	959 (80.5)	459 (19.5)	
2	1386 (83.1)	562 (16.9)	
3	1050 (82.5)	423 (17.5)	
4	455 (84.5)	185 (15.5)	
5	48 (86.4)	25 (13.6)	

* *p* < 0.05 indicates a statistically significant difference in means between participants born in the 50 US States or Washington, D.C., and participants born in other countries. ** Weighted means using constructed 6-Year Mobile Examination sampling weights. † Weighted frequencies using constructed 6-Year Mobile Examination sampling weights. ‡ Significance testing performed through one-way ANOVA and chi-square tests using SAS SURVEYREG and SURVEYFREQ procedures, respectively.

**Table 3:** Individual HEI-2015 total scores * by nativity status.

	US-Native (*n* = 4098)			Non-US-Native (*n* = 1744)		
Food/Nutrient Component	Mean (SE) **	95% CI for Mean	Percent Score	Mean (SE) **	95% CI for Mean	Percent Score
Total vegetables	3.012 (0.043)	2.926, 3.0988	60.2	3.406 (0.061)	3.282, 3.529	68.1
Greens and beans	1.448 (0.053)	1.342, 1.554	29.0	2.338 (0.086)	2.165, 2.512	46.8
Total fruit	1.708 (0.064)	1.578, 1.837	34.2	2.683 (0.065)	2.552, 2.815	53.7
Whole fruit	1.768 (0.077)	1.613, 1.922	35.4	2.826 (0.079)	2.667, 2.986	56.5
Whole grains	2.513 (0.091)	2.33, 2.696	25.1	2.685 (0.156)	2.371, 2.998	26.8
Dairy	5.033 (0.101)	4.831, 5.236	50.3	4.484 (0.104)	4.274, 4.693	44.8
Total protein foods	4.241 (0.03)	4.18, 4.303	84.8	4.388 (0.037)	4.313, 4.463	87.8
Seafood and plant proteins	2.252 (0.052)	2.148, 2.357	45.0	2.958 (0.079)	2.799, 3.118	59.2
Fatty acid ratio	4.861 (0.077)	4.707, 5.015	48.6	5.474 (0.108)	5.256, 5.961	54.7
Sodium	4.226 (0.094)	4.035, 4.416	42.3	4.348 (0.156)	4.033, 4.663	43.5
Refined grains	6.346 (0.084)	6.177, 6.515	63.5	5.092 (0.148)	4.794, 5.39	50.9
Saturated fats	5.375 (0.074)	5.225, 5.524	53.7	6.747 (0.110)	6.525, 6.968	67.5
Added sugars	6.597 (0.115)	6.364, 6.829	66.0	7.806(0.086)	7.633, 7.979	78.1
TOTAL HEI-2015 SCORE	49.38 (0.479)	48.415, 50.344	49.4	55.234 (0.609)	54.008, 56.46	54.44

* Calculated using the Simple Scoring Algorithm. ** Weighted means using constructed 6-Year Dietary Recall Day 1 sampling weights.

**Table 4:** HEI-2015 total scores by nativity status and race/ethnicity.

Race/Ethnicity	Birth Country Mean ± SE		*p* †
	US-Native (*N* = 4098)	Non-US-Native (*N* = 1744)	
Total Unstratified Sample	49.380 ± 0.214	55.234 ± 0.332	<0.0001 *
Mexican American	46.984 ± 0.969	51.200 ± 0.859	<0.0001 *
Other Hispanic	48.177 ± 1.707	53.964 ± 0.796	0.007 *
Non-Hispanic White	49.836 ± 0.601	57.926 ± 1.595	<0.0001 *
Non-Hispanic Black	47.383 ± 0.515	55.427 ± 1.627	<0.0001 *
Non-Hispanic Asian	52.209 ± 1.636	58.108 ± 0.929	0.003 *
Other and/or multiracial	49.867 ± 1.303	57.805 ± 2.934	0.021 *

* *p* < 0.05 indicates a statistically significant difference in the mean HEI-2015 total score of each US-native race/ethnicity and their non-US-native counterparts. † Significance testing performed through one-way ANOVA tests using domain analysis through SAS SURVEYREG procedures.

**Table 5:** Effect of race/ethnicity on age-adjusted and multivariate associations between MetS and nativity °.

Race/Ethnicity	Age-Adjusted OR (95% CI)	*p*	Multivariate OR ^●^ (95% CI)	*p*	Multivariate—HEI OR † (95% CI)	*p*
Mexican American	1.038 (0.72–1.49)	0.835	0.954 (0.64–1.43)	0.817	0.944 (0.61–1.46)	0.795
Other Hispanic	1.107 (0.69–1.75)	0.660	1.036 (0.60–1.79)	0.897	0.974 (0.51–1.86)	0.935
Non-Hispanic White	1.294 (0.79–2.12)	0.301	1.347 (0.80–2.27)	0.254	1.671 (0.88–3.15)	0.110
Non-Hispanic Black	0.409 (0.21–0.80)	0.011 *	0.430 (0.19–0.94)	0.036 *	0.391 (0.17–0.88)	0.024 *
Non-Hispanic Asian	0.894 (0.45–1.77)	0.745	0.879 (0.45–1.72)	0.702	1.289 (0.55–2.99)	0.548
Other and/or multiracial	0.707 (0.16–3.19)	0.645	0.626 (0.23–1.72)	0.357	1.089 (0.37–3.23)	0.875

° Associations analyzed through logistic regression modeling using SAS SURVEYLOGISTIC procedures. * *p* < 0.05 indicates a statistically significant difference in odds of developing MetS compared to US-native participants of the same race/ethnicity. ^●^ Adjusted for age, sex, race, and education level. † Adjusted for age, sex, race, education level, and HEI-2015 total score quintiles.

**Table 6:** Multivariate-adjusted associations of MetS and clinical components and nativity °.

Race/Ethnicity	Individual MetS Clinical Components OR (95% CI)				
	Waist Circumference	Triglycerides	HDL Cholesterol	Fasting Plasma Glucose	Blood Pressure
Overall	0.588 (0.497–0.696) *	1.314(1.049–1.646) *	0.832(0.612–1.130)	1.336(1.044–1.709)	1.003 (0.829–1.213)
Mexican American	0.720 (0.458–1.136)	1.366 (0.884–2.112)	0.856 (0.500–1.466)	1.286(0.688–2.407)	0.758 (0.533–1.079)
Other Hispanic	0.352 (0.191–0.648) *	0.714 (0.409–1.245)	0.888 (0.458–1.722)	0.842(0.499–1.576)	0.979 (0.528–1.816)
Non-Hispanic White	0.722 (0.388–1.345)	1.308 (0.665–2.574)	1.262(0.495–3.221)	1.327 (0.812–2.169)	1.104 (0.579–2.107)
Non-Hispanic Black	0.544 (0.325–0.943) *	0.365 (0.080–1.663)	0.633(0.349–1.149)	0.773 (0.403–1.485)	0.521 (0.285–0.953)
Non-Hispanic Asian	1.175 (0.491–2.815)	0.974 (0.444–2.137)	0.308 (0.135–0.700) *	1.635 (0.644–4.151)	1.137 (0.632–2.786)
Other and/or multiracial	2.120 (0.339–13.243)	4.970 (1.146–21.547) *	0.859 (0.266–2.766)	0.352 (0.085–1.454)	0.652 (0.266–2.582)

° Associations analyzed through logistic regression using SAS SURVEY LOGISTIC procedures and are adjusted for age, race, sex, education level, and HEI-2015 total score quintiles. * *p* < 0.05 indicates a statistically significant difference in odds of having a clinical measurement that meets the ATP III MetS identification criteria compared to US-native participants of the same race/ethnicity.

## Data Availability

NHANES data are publicly available through the Centers for Disease Control and Prevention.
